# Parental substance use disorder and risk of intellectual disability in offspring in Sweden: a national register study

**DOI:** 10.1016/j.eclinm.2023.102170

**Published:** 2023-08-30

**Authors:** Lotfi Khemiri, Ralf Kuja-Halkola, Henrik Larsson, Agnieszka Butwicka, Magnus Tideman, Brian M. D'Onofrio, Antti Latvala, Paul Lichtenstein

**Affiliations:** a Department of Medical Epidemiology and Biostatistics, Karolinska Institutet, Stockholm, Sweden; b Department of Clinical Neuroscience, Centre for Psychiatry Research, Karolinska Institutet, Stockholm, Sweden; c School of Medical Sciences, Örebro University, Örebro, Sweden; d Institute of Clinical Medicine, University of Oslo, Oslo, Norway; e Division of Mental Health Services, Akershus University Hospital, Lørenskog, Norway; f Department of Biostatistics and Translational Medicine, Medical University of Lodz, Lodz, Poland; g Department of Social Sciences, Marie Cederschiöld University, Stockholm, Sweden; h School of Health and Welfare, Halmstad University, Halmstad, Sweden; i Department of Psychological and Brain Sciences, Indiana University, Bloomington, IN, USA; j Institute of Criminology and Legal Policy, University of Helsinki, Helsinki, Finland

**Keywords:** Intellectual disability, Mental retardation, Parental substance use disorder, Parental alcohol use disorder, Parental drug use disorder

## Abstract

**Background:**

Intellectual disability (ID) is a disorder with unknown aetiology in many cases. Maternal alcohol use is a known risk factor for ID, but less is known about the importance of maternal and paternal substance use disorder (SUD) and risk of ID in offspring.

**Methods:**

Data from multiple nationwide registers were used to create a cohort of children born from January 01, 1978 to December 31, 2002. All participants were born in Sweden, had available parental identification information and did not emigrate or die before age 12 (n = 1,940,820). Logistic regression modelling was performed with exposure defined as having a parent who received any SUD diagnosis, including alcohol use disorder (AUD) and drug use disorder (DUD). The outcome was registration of diagnosis of any form of ID. First, we analysed the risk of ID if parental SUD was registered prior to childbirth with stepwise adjustment of multiple covariates. Second, the effect of timing of SUD diagnosis in relation to childbirth was analysed.

**Findings:**

Of 37,410 offspring with parental SUD registered prior to birth, 3.0% (n = 1110) had any form of ID compared to 1.2% (n = 23,168) of those 1,903,410 individuals without parental SUD prior birth. Parental SUD prior birth was associated with an increased risk of any form of ID (Odds Ratio [OR]: 2.3 [2.2–2.5]), with ORs similar for maternal (OR: 2.3 [2.1–2.5]) and paternal SUD (OR: 2.3 [2.1–2.5]). These ORs were reduced but remained statistically significant after adjusting for parental education, migration, psychiatric comorbidity, and co-parent SUD (OR parental SUD: 1.6 [1.5–1.8]; OR maternal SUD: 1.4 [1.2–1.5]; OR paternal SUD: 1.6 [1.5–1.7]). Parental SUD was associated with increased risk of ID in offspring irrespective of timing of diagnosis, but if mothers or fathers were diagnosed with AUD during pregnancy (OR maternal AUD: 5.0 [3.1–8.2]; OR paternal AUD: 2.8 [2.2–3.6]), the risk was significantly greater than if the AUD diagnosis was first registered after childbirth (OR maternal AUD: 1.9 [1.8–2.0]; OR paternal AUD: 1.6 [1.6–1.7]).

**Interpretation:**

Both paternal and maternal SUD were associated with an increased risk of ID in offspring, with greatest risk observed when AUD was diagnosed during pregnancy. Possible mechanisms may involve shared genetic and environmental factors, including toxic effects from alcohol intake. These findings have clinical implications in suggesting that parental SUD in either parent represents a possibly modifiable risk factor to consider when developing prevention, diagnostics and treatment programs for children with ID.

**Funding:**

10.13039/501100004348Stockholm County Council, the Research Council of the Swedish Alcohol Retailing Monopoly, Fredrik and Ingrid Thurings stiftelse, 10.13039/501100002341Academy of Finland, the 10.13039/501100004359Swedish Research Council and the Swedish Research Council for Health, Working Life and Welfare, Nordforsk by the Nordic Council of Ministers and the Polish Medical Research Agency.

**Research in context**

**Evidence before this study**

To investigate the association between parental substance use disorder (SUD) and intellectual disability (ID) in offspring we searched Medline for systematic reviews published until 1 January 2023. We utilized the search terms “systematic review” or “review” and “parental risk” or “risk factor” or “prenatal risk” or “perinatal” or “neonatal” or “parental substance use disorder” or “parental alcohol use disorder” or “parental drug use disorder” (as well as maternal/paternal) or “alcohol during pregnancy” or “drug use during pregnancy” and “mental retardation” or “intellectual disability” or “developmental disability”. The most recent and extensive systemic review was identified (Leonard et al., 2022), which included 58 studies presenting multiple risk factors such as low socioeconomic status, minority status, maternal mental illness and maternal alcohol use. Importantly, besides advanced paternal age and paternal epilepsy, no other paternal risk factors were identified. Taken together, previous research has identified multiple maternal risk factors for ID, but little is known regarding maternal and paternal SUD, including alcohol use disorder (AUD) and drug use disorder (DUD), and risk of ID in offspring.

**Added value of this study**

To the best of our knowledge, the current study is the first to utilize large-scale national registry-data to investigate the association between parental SUD and risk of ID in offspring. The main finding is that at the population level, both mothers and fathers with SUD diagnosed before childbirth have children with an increased risk of being diagnosed with ID of all severity forms. Importantly, the risk was evident for both alcohol and drug related diagnoses in both parents, while parental alcohol abuse diagnosed during pregnancy conferred the greatest risk of ID in offspring. The underlying mechanism is not known, but may involve genetic and environmental factors, including toxic effects from alcohol intake on foetal development.

**Implications of all the available evidence**

These findings suggest that parental SUD of all forms in both fathers and mothers constitute a risk factor for developing ID. Parental SUD may thus represent one out of multiple risk factors underlying ID of unknown aetiology. This has implications for health care and social services working with SUD parents and children with ID. Prevention efforts, education of health care staff and public health recommendations have for decades focused on mothers with alcohol related problems. The current study however highlights the importance of targeting not only mothers but also fathers with SUD when developing tools of prevention, diagnostics and treatment efforts for children with ID.

## Introduction

Intellectual disability (ID) is a neurodevelopmental disorder with a global prevalence of approximately 1%.^1^ It is characterized by low general intelligence and difficulties with adaptive behaviour and impairments in cognitive, motor, language and social skills.^2^ Individuals with ID exhibit high levels of psychiatric symptoms^3^ and worse physical health,^4^ including excess premature mortality.^5^ The disorder is also associated with great societal economic costs.^6^ While ID can be caused by specific genetic syndromes e.g., Trisomy 21, Fragile X and Prader–Willi,^7^ the aetiology remains unknown for the majority of ID cases.^8^

Substance use disorders (SUDs) are common psychiatric disorders responsible for great morbidity and mortality worldwide.^9^ Parental SUD is associated with several negative psychosocial outcomes,^10^ including reduced general cognitive ability in offspring,^11^ but little is known regarding the role of parental SUD and the more severe phenotype of ID. While some studies have suggested shared genetic factors underlying the association between SUD and lower cognitive abilities,^12^^,^^13^ there is also a plausible mechanism of direct causal effect on cognitive development via uterine effects from substance intake on the developing foetal brain. The best characterized syndrome is Foetal Alcohol Spectrum Disorders (FASD), in which maternal alcohol intake during pregnancy results in congenital malformations as well as motor and cognitive impairments including ID in offspring.^14^ While clinical case studies do provide valuable evidence,^15^ few studies have utilized large-scale population-based epidemiological approaches to investigate the association between parental SUD and offspring ID. In an Australian population sample, O'Leary and colleagues found three-fold elevated risk of ID in offspring of mothers with any alcohol dependence diagnosis during pregnancy,^16^ but this study did not investigate the putative role of fathers SUD status. Two systematic reviews have been conducted on risk factors for the development of ID: Huang and colleagues focused on prenatal, perinatal and neonatal risk factors and describe ten maternal prenatal risk factors for ID, including advanced maternal age, alcohol use, tobacco and diabetes.^17^ More recently, Leonard and colleagues performed a systematic review of broader variable categories and identified multiple risk factors such as low socioeconomic status, minority status, maternal mental illness and maternal alcohol abuse.^18^ While observational data cannot establish the causal pathways, previously observed risk factors are likely multifactorial and represent indices of both environmental and genetic risk factors. However, identifying novel risk factors could improve early detection of ID in families at risk, as well as generating hypotheses regarding causal mechanisms.

Besides advanced paternal age and paternal epilepsy, no other paternal risk factors have been described,^18^ highlighting a gap in the research literature which has been focused on substance use in mothers rather than fathers. It is understandable that much research has been investigating the role of maternal substance use given the risk of in utero effects, but if SUD in fathers also increases ID risk it would be possible to improve identification of vulnerable children at risk. In addition, such a putative relation between paternal substance use and ID could shed light on novel hypotheses regarding the aetiology of ID, such as shared genetic factors between SUD and ID or a possible role of substance toxic effects on male germ line cells. To the best of our knowledge, no previous study has investigated the role of paternal and maternal SUD including alcohol use disorder (AUD) and drug use disorder (DUD) on risk of offspring ID. In addition, little is known regarding how timing of diagnosis is associated with ID risk, and whether the risk is different for milder compared to more severe forms of ID. Since parental SUD represents an identifiable and putatively preventable risk factor for a rare outcome such as offspring ID, this research question is suitable to investigate using large-scale national register data.

The aim of the current study was thus to investigate how maternal and paternal SUDs are associated with offspring ID of different severity, by utilizing Swedish national registries in a large-scale population-based study. Furthermore, the timing of parental SUD diagnosis was analysed in an attempt to investigate putative effects from foetal exposure of substance intake during pregnancy. The main hypotheses were that parental SUD would increase risk of ID in offspring and that this risk would be greatest if diagnosis was registered during pregnancy.

## Methods

### National registries

All people living in Sweden are given a unique personal identification number, allowing linkage between different national registries. The current study utilized data from the National Patient Register, the Multi-Generation Register, the Migration Register, the Total Population Register and the Cause of Death Register described in detail in the supplementary material. The register-linkage has been approved by the Regional Ethical Review Board in Stockholm, Sweden and the requirement of informed consent was waived given the use of register data. The study adheres to the STROBE reporting guidelines.

### Study cohort

**Fig. 1:**
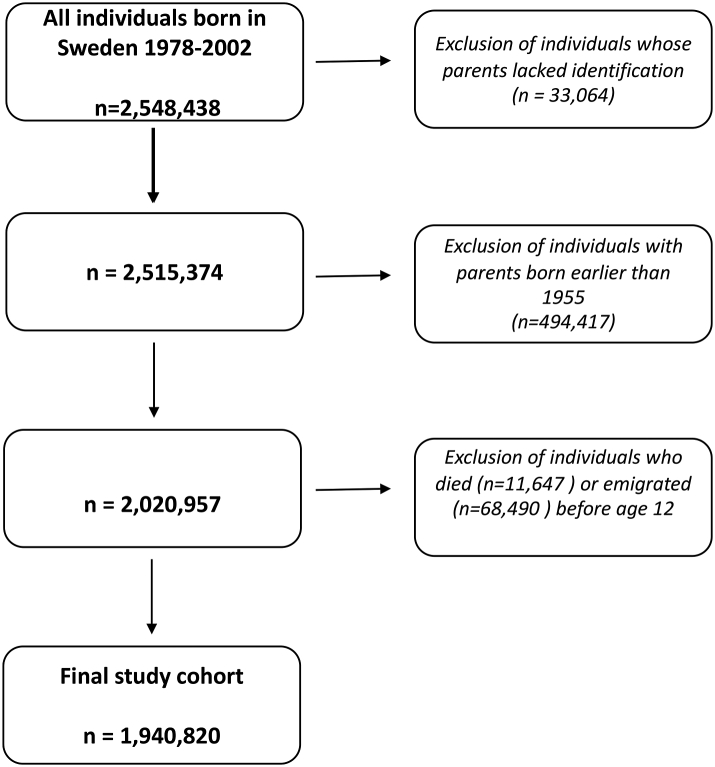
Study diagram illustrating the creation of the study cohort.

The study population was all individuals born in Sweden from January 01, 1978 to December 31, 2002 (n = 2,548,438). We excluded individuals whose parents could not be identified (n = 33,064), those whose both parents were born earlier than 1955 and therefore not fully covered by the patient register in adulthood (n = 494,417), and those who died (n = 11,647) or emigrated (n = 68,490) before age 12. This resulted in a final study cohort of 1,940,820 individuals (Fig. 1 illustrates the study flow diagram). The earliest birth year of 1978 allowed for a minimum of 5 years of minimum register coverage for parents prior to any offspring birth. The total follow-up time period was from January 01, 1978 to December 31, 2020.

### Exposure

Parental SUD was operationalized as any maternal or paternal SUD diagnosis (ICD-8:291, 303, 304; ICD-9: 291, 292, 303, 304, 305, 980; ICD-10:F10-F19 (except F17), X41, X42, X45, X61, X62, X65, Y11, Y12, Y15, T40, T51) registered in the National Patient Register. Table S1 describes the SUD diagnoses in detail, further divided into AUD and DUD. We chose to include SUD diagnoses including the intoxication diagnoses related to self-harm, given the research question related to putative effects from substance intake on foetal development. In addition, the substance abuse in these cases resulted in health care utilization, suggestive of hazardous use and/or substance related physical or psychological health problems which are both diagnostic criteria of SUD.^2^

### Outcome

ID was defined as receiving any ID diagnosis (ICD-8:310-315; ICD-9: 317-319; ICD-10: F70-73, F78-79) during follow-up. The ID group was further subdivided into mild-unspecified ID and severe-moderate ID based on the most severe type of ID diagnosis ever registered in the NPR. Table S2 describes detailed information regarding the ID diagnoses utilized.

### Covariates

Offspring sex, birth year, and mother's and father's age at birth of a child were included as covariates in the main model. In addition, we further adjusted for additional covariates: highest level of parental education, parental immigration, and psychiatric co-morbidity (schizophrenia, bipolar disorder, depression and anxiety disorders registered before birth of offspring). The covariates and psychiatric diagnostic codes are presented in detail in the supplementary material (Table S3). The rationale for adjusting for birth year was due to the possibility of changes in definitions and diagnostic procedures regarding both parental SUD and ID in offspring across time. We adjusted for sex since ID is more common in men compared women, possibly due to increased frequency of risk genes on the X chromosome.^19^ Remaining covariates were included since these have been implicated as risk factors for ID in previous research.^17^^,^^18^

### Statistical analyses

Two main analyses are presented in the manuscript. First, parental SUD registered prior to childbirth and risk of ID in offspring was analysed. Second, we investigated how the timing of parental SUD diagnosis registration was related to ID risk in offspring. Separate analyses were performed for maternal SUD and paternal SUD, and further for parental AUD and DUD separately.

### Association between parental SUD before birth and offspring ID

First, the association between parental SUD prior to offspring birth and ID in offspring was analysed. Descriptive statistics was done by calculating prevalence of different forms of ID and sociodemographic variables with 95% confidence intervals (CI) in offspring who had parental/maternal/paternal SUD diagnosed prior to birth of offspring. We analysed the association between parental SUD and ID in offspring using logistic regression models, using three models with increased adjustment of covariates as follows: Model 1 adjusted for offspring sex, offspring birth year, and age of mother and father at offspring birth. Model 2 included same covariates as model 1 but further adjusted for parental highest education and migration status. Model 3 included same covariates as model 2 but further adjustment of parental psychiatric co-morbidity and the co-parents SUD status (in the maternal/paternal analyses). The rationale for restricting parental SUD to those with a diagnosis registered prior to birth of offspring in this analysis, was to minimize the risk of reverse causation and confirm that the onset of SUD was indeed before the birth of offspring. Because ID in general is congenital, we chose not to include the timing of ID diagnosis in the analyses as this likely reflects differences in symptoms, severity, and healthcare accessibility, rather than the onset of the disorder.

### Timing of parental SUD diagnosis in relation to offspring ID risk

Since timing of first SUD diagnosis is not necessarily related to onset of substance related problems, we wanted to further investigate the effect of timing of parental SUD diagnosis in relation to risk of ID in offspring. Logistic regression models were again fitted, but employing three different definitions of the exposure variable: 1) First SUD diagnosis registered before pregnancy (but no diagnosis during pregnancy); 2) SUD diagnosis during pregnancy (defined as registration any time during the 9 months prior to birth of child); 3) First SUD diagnosis registered after birth of offspring. In this analysis we only report one model and thus adjust for offspring sex, offspring birth year, and age of mother and father at offspring birth. We chose to focus on the pregnancy period primarily in order to detect putative in utero effects in mothers, and we used the identical time periods for fathers in order to keep the maternal/paternal analyses comparable. In addition, during pregnancy families in general establish contact with maternity care services. There is thus also a clinical rationale to focus on this period since if SUD diagnosis (in any parent) would increase ID risk it would have specific clinical implications for maternity care services.

### Sensitivity analyses

Several sensitivity analyses were performed where the first main analysis of parental SUD prior to childbirth and risk of ID model 1 was repeated but with the following adjustments: First, in order to investigate whether intoxication diagnoses were driving the observed associations we repeated the analysis separately for non-intoxication diagnoses and intoxication diagnoses as exposure. Second, since previous studies have shown significant heritability of ID, the analysis was repeated with adjustment for parental lifetime ID diagnoses. Third, the analysis was repeated excluding all offspring with any diagnosed chromosomal disorders (ICD 9:758; ICD-10: Q90-Q99) in order to exclude cases where the cause of ID was known. Fourth, we calculated the probability of having a mother with SUD if you have a father with SUD by performing a two-way chi square test for maternal/paternal SUD including AUD and DUD. Finally, to investigate cohort effects (e.g., changes in incidence or diagnostic/registration practices across time) we repeated the analysis without any restrictions regarding parental birth year, i.e., we included children with parents born earlier than 1955. We also subdivided the original cohort into a young cohort (born 1991–2002) and old cohort (born 1978–1990), and calculated prevalence rates of parental SUD and ID in offspring, as well as repeating the analysis of parental SUD prior to childbirth and ID in both cohorts separately. Lastly, we repeated the same analysis but excluded all individuals who died or emigrated during follow-up, in order to ensure that our results were not affected by those individuals who because of death or emigration did not experience the same time period at risk.

SAS (version 9.4, SAS Institute Inc.) was used for data management. All statistical analyses were performed using Stata (version 17.0, Statacorp LLC). Due to the non-independence of offspring siblings, all statistical analyses used a cluster robust sandwich estimator for standard errors, based on the maternal personal identification number.

### Role of the funding source

The funder of the study had no role in study design, data collection, data analysis, data interpretation, or writing of the report. Authors LK, RKH and PL had access to the dataset and final responsibility for the decision to submit for publication.

## Results

### Descriptive results

The full descriptive results are presented in Table 1. Of offspring with parental SUD prior to birth (n = 37,410), 3.0% had any form of ID compared to 1.2% of those without parental SUD (n = 1,903,410) with similar levels in those with maternal (3.1%) and paternal (2.9%) SUD. In the group with parental SUD prior birth, 0.6% had severe-moderate ID and 2.4% had mild-unspecific ID compared to 0.3% and 0.9% in the unexposed group. The parents with SUD prior to childbirth had lower education, three-to fourfold higher rates of psychiatric co-morbidity and slightly lower rate of immigrant background. During the follow-up period, 12,491 individuals died, 84,996 emigrated and 24,278 received an ID diagnosis. After taking into account whatever came first (end of study follow-up, death, emigration, ID diagnosis), the mean follow-up period, i.e., time at risk, was 28.1 years (standard deviation: 6.5; interquartile range: 9.7).

**Table 1:** Intellectual disability (ID) and sociodemographic characteristics in 1,940,820 individuals born in Sweden 1978–2002 (males n = 997,085; females n = 943,735), with and without parental substance use disorder (SUD) diagnosed before birth of offspring. Values in brackets are 95% confidence intervals.

Full sample (n = 1,940,820)	No parental SUD (n = 1,903,410)	SUD in any parent (n = 37,410)	Maternal SUD (n = 13,582)	Paternal SUD (n = 26,142)
**Any ID**				
No.	23,168	1110	415	768
%	1.2 [1.2–1.2]	3.0 [2.8–3.1]	3.1 [2.7–3.4]	2.9 [2.7–3.1]
**Severe-moderate ID**				
No.	5886	229	85	156
%	0.3 [0.3–0.3]	0.6 [0.5–0.7]	0.6 [0.5–0.8]	0.6 [0.5–0.7]
**Mild-unspecified ID**				
No.	17,282	881	330	612
%	0.9 [0.9–0.9]	2.4 [2.2–2.5]	2.4 [2.2–2.7]	2.3 [2.2–2.5]
**Fathers education, mean**^a^, ^b^	3.7 [3.7–3.7]	3.0 [3.0–3.1]	3.2 [3.1–3.2]	3.0 [2.9–3.0]
**Mothers education, mean**^a^^,^^c^	4.1 [4.1–4.1]	3.5 [3.5–3.5]	3.3 [3.3–3.3]	3.5 [3.5–3.6]
**Fathers psychiatric disorder**^d^				
No.	138,566	10,900	2419	9270
%	7.3 [7.2–7.3]	29.1 [28.7–29.6]	17.8 [17.2–18.5]	35.5 [34.9–36.0]
**Mothers psychiatric disorder**^d^				
No.	212,090	11,649	6381	6453
%	11.1 [11.1–11.2]	31.1 [30.7–31.6]	47.0 [46.1–47.8]	24.7 [24.2–25.2]
**Father immigrant**				
No.	270,982	4909	1983	3218
%	14.2 [14.2–14.3]	13.1 [12.8–13.5]	14.6 [14.0–15.2]	12.3 [11.9–12.7]
**Mother immigrant**				
No.	253,495	4389	1444	3180
%	13.3 [13.3–13.4]	11.7 [11.4–12.1]	10.6 [10.1–11.2]	12.2 [11.8–12.6]

a Parental education data was extracted as a scale from 1 (did not complete mandatory school) to 7 (post-graduate education completed) and reported as mean value.

b Missing data on paternal education resulted in total sample size of n = 1,931,314.

c Missing data on maternal education resulted in total sample size of n = 1,937,711.

d Lifetime diagnosis of schizophrenia, bipolar disorder, depressive disorder or anxiety disorder.

### Association between parental SUD before birth and offspring ID

The full results of the first main analysis of parental SUD registered prior to childbirth and risk of ID are presented in Table 2. Parental SUD prior to birth was associated with a significantly increased risk of any form of ID in offspring (Odds Ratio [OR]: 2.3 [2.2–2.5]), and the ORs were similar for both maternal (OR: 2.3 [2.1–2.5]) and paternal SUD (OR: 2.3 [2.1–2.5]). Slightly greater OR was observed for mild-unspecified ID (2.4) compared to severe-moderate ID (1.9). In model 1, similar overall results were found for parental AUD and DUD on all ID forms.

**Table 2:** Parental substance use disorder (SUD), including alcohol use disorder (AUD) and drug use disorder (DUD), before childbirth as predictors of any intellectual disability (ID), severe-moderate ID and mild-unspecified ID using logistic regression modelling.

Full sample (n = 1,940,820)^a^	SUD in any parent (n = 37,410)	Maternal SUD (n = 13,582)	Paternal SUD (n = 26,142)
**Any ID**			
No. cases (%)	1110 (3.0)	415 (3.1)	768 (2.9)
Model 1^b^	2.3 [2.2–2.5]	2.3 [2.1–2.5]	2.3 [2.1–2.5]
Model 2^c^	1.8 [1.7–2.0]	1.7 [1.6–1.9]	1.8 [1.7–2.0]
Model 3^d^	1.6 [1.5–1.8]	1.4 [1.2–1.5]	1.6 [1.5–1.7]
**Severe-moderate ID**			
No. cases (%)	229 (0.6)	85 (0.6)	156 (0.6)
Model 1^b^	1.9 [1.6–2.1]	1.9 [1.5–2.4]	1.8 [1.5–2.1]
Model 2^c^	1.6 [1.4–1.9]	1.6 [1.3–2.0]	1.6 [1.3–1.9]
Model 3^d^	1.5 [1.3–1.8]	1.3 [1.1–1.7]	1.4 [1.2–1.7]
**Mild-unspecified ID**			
No. cases (%)	881 (2.4)	330 (2.4)	612 (2.3)
Model 1^b^	2.4 [2.3–2.6]	2.4 [2.1–2.7]	2.4 [2.2–2.6]
Model 2^c^	1.9 [1.7–2.0]	1.7 [1.6–2.0]	1.9 [1.7–2.1]
Model 3^d^	1.6 [1.5–1.8]	1.3 [1.2–1.5]	1.6 [1.5–1.8]
	**AUD in any parent (n** = **28,245)**	**Maternal AUD (n** = **9202)**	**Paternal AUD (n** = **20,113)**

**Any ID**			
No. cases (%)	882 (3.1)	292 (3.2)	636 (3.2)
Model 1^b^	2.4 [2.2–2.6]	2.3 [2.1–2.6]	2.5 [2.3–2.7]
Model 2^c^	1.9 [1.8–2.1]	1.8 [1.6–2.0]	2.0 [1.8–2.2]
Model 3^d^	1.7 [1.6–1.8]	1.4 [1.2–1.6]	1.7 [1.6–1.9]
**Severe-moderate ID**			
No. cases (%)	177 (0.6)	58 (0.6)	127 (0.6)
Model 1^b^	1.9 [1.6–2.2]	1.9 [1.4–2.5]	1.9 [1.6–2.3]
Model 2^c^	1.7 [1.4–2.0]	1.6 [1.2–2.1]	1.7 [1.4–2.0]
Model 3^d^	1.5 [1.3–1.8]	1.3 [1.0–1.8]	1.5 [1.3–1.9]
**Mild-unspecified ID**			
No. cases (%)	705 (2.5)	234 (2.5)	509 (2.5)
Model 1^b^	2.6 [2.4–2.8]	2.4 [2.1–2.8]	2.6 [2.4–2.9]
Model 2^c^	2.0 [1.8–2.1]	1.8 [1.6–2.1]	2.1 [1.9–2.3]
Model 3^d^	1.7 [1.6–1.9]	1.4 [1.2–1.6]	1.8 [1.6–2.0]
	**DUD in any parent (n** = **15,650)**	**Maternal DUD (n** = **6259)**	**Paternal DUD (n** = **10,540)**

**Any ID**			
No. cases (%)	444 (2.8)	192 (3.1)	279 (2.6)
Model 1^b^	2.2 [2.0–2.4]	2.3 [2.0–2.7]	2.0 [1.8–2.3]
Model 2^c^	1.7 [1.5–1.8]	1.7 [1.4–2.0]	1.6 [1.4–1.8]
Model 3^d^	1.4 [1.3–1.6]	1.3 [1.1–1.5]	1.3 [1.1–1.5]
**Severe-moderate ID**			
No. cases (%)	90 (0.6)	37 (0.6)	58 (0.6)
Model 1^b^	1.7 [1.4–2.2]	1.8 [1.3–2.5]	1.7 [1.3–2.2]
Model 2^c^	1.5 [1.2–1.8]	1.5 [1.0–2.0]	1.4 [1.0–1.8]
Model 3^d^	1.3 [1.0–1.6]	1.2 [0.8–1.7]	1.2 [0.9–1.6]
**Mild-unspecified ID0**			
No. cases (%)	354 (2.3)	155 (2.5)	221 (2.1)
Model 1^b^	2.3 [2.0–2.6]	2.5 [2.1–3.0]	2.1 [1.8–2.4]
Model 2^c^	1.7 [1.5–1.9]	1.7 [1.4–2.1]	1.6 [1.4–1.8]
Model 3^d^	1.4 [1.3–1.6]	1.3 [1.1–1.6]	1.3 [1.1–1.5]

Values for each model are presented as Odds Ratios (OR) with 95% confidence intervals in brackets. Standard errors were adjusted for the clustering of siblings.

a In Model 2 and Model 3, the sample size was n = 1,928,488 due to missing information on parental education in 12,332 individuals.

b Model 1 is the main analysis reported in the manuscript including adjustment for sex, birth year and age at birth in mother and father.

c Model 2 same as Model 1 with further adjusted for parental highest education and migration status.

d Model 3 same as Model 2 with further adjusted for parental psychiatric disorders (schizophrenia, bipolar disorder, depressive disorder or anxiety disorder registered before childbirth) and the co-parents SUD/AUD/DUD status (in the maternal/paternal analyses).

After stepwise adjustment of the additional covariates of parental education, migration and psychiatric co-morbidity and co-parental SUD, the ORs for parental SUD, AUD and DUD on risk of any ID in offspring were reduced by approximately 30% but remained overall statistically significant. A similar pattern was observed for mild-unspecified ID which constituted the majority of ID cases. Exceptions were noted for moderate-severe ID, for which maternal AUD and parental DUD were not statistically significant after full adjustment. It should however be emphasized that there were only 229 cases of severe-moderate ID in the entire parental SUD group, and the subgroup analyses should thus be interpreted cautiously. Regarding co-parent SUD status, as expected it increased risk of ID both when the co-parent with SUD was a father (1.6 [1.5–1.7]) or mother (1.4 [1.2–1.5]). Among the other covariates in the parental SUD and any ID analysis, parental psychiatric comorbidity (OR mother: 1.9 [1.7–2.1]; OR father: 1.6 [1.4–1.8]) increased risk of ID, while female sex (OR: 0.8 [0.7–0.8]) and higher parental education (OR mother: 0.8 [0.7–0.8]; OR father: 0.9 [0.9–0.9]) reduced the ID risk. The other covariates did not explain much variance and were all close to one (Table S4 presents the ORs of all covariates in model 3).

### Timing of parental SUD diagnosis in relation to offspring ID risk

The full results of the second main analysis of timing of parental SUD diagnosis and ID risk in offspring are presented in Table 3. Overall, parental SUD, irrespective of the timing of diagnosis, was associated with a statistically significantly increased OR for all forms of ID in offspring, with similar ORs as in the first main analysis of parental SUD diagnosed prior to childbirth. The confidence intervals were in general overlapping for the different time periods–but a notable observation was that for parental SUD diagnosed only before pregnancy (OR any ID: 2.3 [2.1–2.4]) or during pregnancy (OR any ID: 2.6 [2.2–3.2]) there was a statistically significant risk increase compared to if the parent received the SUD diagnosis after birth (OR any ID: 1.8 [1.7–1.9]). The same pattern was found for paternal and maternal SUD across all severity forms of ID.

**Table 3:** Different time periods of paternal and maternal substance use disorder (SUD) diagnosis registration as predictors of any intellectual disability (ID), severe-moderate ID and mild-unspecified ID using logistic regression modelling with adjustment for sex, birth year and age at birth in mother and father.

Full sample (n = 1,940,820)	Parental SUD only before pregnancy (n = 34,208)	Parental SUD during pregnancy (n = 3268)	Parental SUD only after birth (n = 189,568)
**Any ID**			
No. cases (%)	1002 (2.9)	111 (3.4)	3843 (2.0)
OR	2.3 [2.1–2.4]	2.6 [2.2–3.2]	1.8 [1.7–1.9]
**Severe-moderate ID**			
No. cases (%)	205 (0.6)	26 (0.8)	818 (0.4)
OR	1.8 [1.6–2.1]	2.5 [1.7–3.6]	1.5 [1.4–1.6]
**Mild-unspecified ID**			
No. cases (%)	797 (2.3)	85 (2.6)	3025 (1.6)
OR	2.4 [2.2–2.6]	2.7 [2.2–3.3]	1.9 [1.8–2.0]
	**Maternal SUD before pregnancy (n** = **12,907)**	**Maternal SUD during pregnancy (n** = **694)**	**Maternal SUD only after birth (n** = **79,207)**

**Any ID**			
No. cases (%)	388 (3.0)	29 (4.2)	1807 (2.3)
OR	2.2 [2.0–2.5]	3.2 [2.2–4.7]	1.9 [1.8–2.0]
**Severe-moderate ID**			
No. cases (%)	79 (0.6)	7 (1.0)	370 (0.5)
OR	1.8 [1.5–2.3]	3.0 [1.4–6.3]	1.6 [1.4–1.8]
**Mild-unspecified ID**			
No. cases (%)	309 (2.4)	22 (3.2)	1437 (1.8)
OR	2.3 [2.1–2.6]	3.2 [2.1–4.9]	2.0 [1.9–2.2]
	**Paternal SUD before pregnancy (n** = **23,492)**	**Paternal SUD during pregnancy (n** = **2700)**	**Paternal SUD only after birth (n** = **122,898)**

**Any ID**			
No. cases (%)	682 (2.9)	87 (3.2)	2405 (2.0)
OR	2.3 [2.1–2.4]	2.5 [2.0–3.1]	1.7 [1.6–1.8]
**Severe-moderate ID**			
No. cases (%)	136 (0.6)	21 (0.8)	522 (0.4)
OR	1.8 [1.5–2.1]	2.4 [1.6–3.7]	1.4 [1.3–1.6]
**Mild-unspecified ID**			
No. cases (%)	546 (2.3)	66 (2.4)	1883 (1.5)
OR	2.4 [2.2–2.6]	2.5 [2.0–3.2]	1.8 [1.7–1.9]

Values are presented as Odds Ratios (OR) with 95% confidence intervals in brackets.

Standard errors were adjusted for the clustering of siblings.

**Fig. 2:**
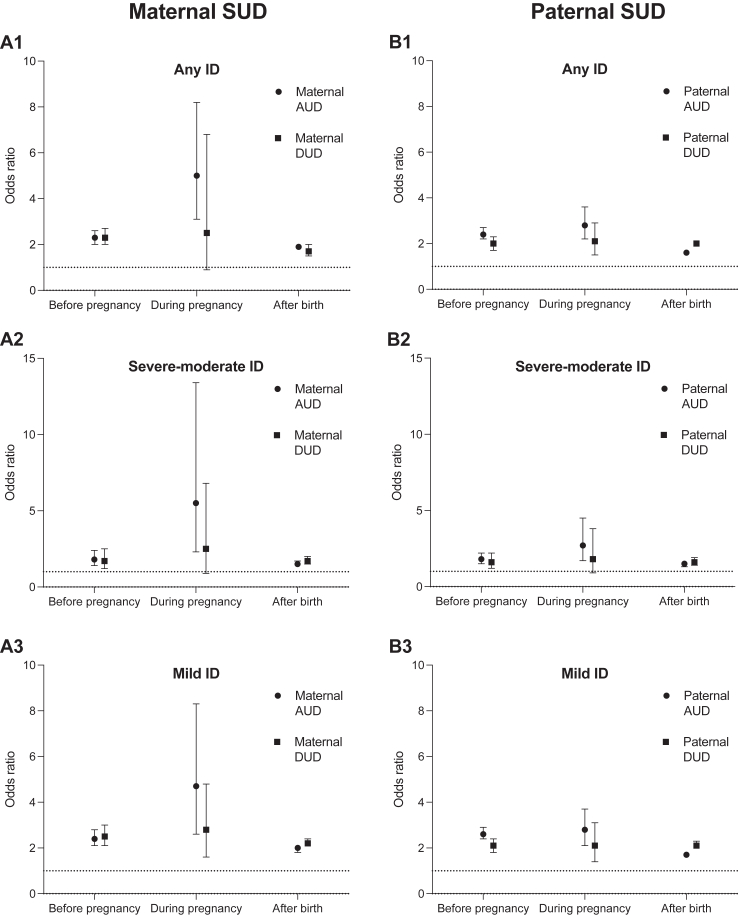
Paternal and maternal substance use disorder (SUD) diagnosis, subdivided into alcohol use disorder (AUD) and drug use disorder (DUD), as predictors of any intellectual disability (ID), severe-moderate ID and mild-unspecified ID. Logistic regression modelling was employed for different time periods based on when SUD diagnosis was registered (before pregnancy, during pregnancy or after birth of offspring). Analyses were adjusted for sex, birth year and age at birth in mother and father. The y axis for severe-moderate ID ranges from 0 to 15, while this range is 0–10 for any ID and mild ID. Values are presented as Odds Ratios (OR) with 95% confidence intervals error bars. Standard errors were adjusted for the clustering of siblings.

Fig. 2 presents the ORs for maternal and paternal AUD/DUD and risk of ID in offspring. While keeping in mind the wide CIs and low number of ID cases, maternal AUD diagnosed during pregnancy was associated with greater OR for any form of ID (OR: 5.0 [3.1–8.2]) compared to if diagnosis was registered prior to pregnancy (OR: 2.3 [2.0–2.6]) or after childbirth (OR: 1.9 [1.8–2.0]). In addition, maternal AUD during pregnancy had a significantly greater OR for severe-moderate ID (OR: 5.5 [2.3–13.4] and mild-unspecified ID (OR: 4.7 [2.6–8.3] compared to if diagnosis was registered after birth (severe-moderate ID OR: 1.5 [1.4–1.7]; mild-unspecified ID OR: 2.0 [1.8–2.1]). The same pattern in relation the pregnancy period was however not observed for maternal DUD, where the OR during pregnancy was only statistically significant for mild ID, and with overall overlapping CIs across the different time periods. For the fathers, all ORs were statistically significantly different from one across the time periods, with the exception of paternal DUD during pregnancy and severe-moderate ID. Notable however, and similar to the mothers, the ORs for paternal AUD during pregnancy (Any ID: 2.8 [2.2–3.6]; Severe-moderate ID: 2.7 [1.7–4.5]; Mild-unspecified ID: 2.8 [2.1–3.7]) were significantly greater than if the paternal AUD diagnoses were first registered after birth (Any ID: 1.6 [1.6–1.7]; Severe-moderate ID: 1.5 [1.3–1.6]; Mild-unspecified ID: 1.7 [1.6–1.8]). Again, no such pattern was observed for paternal DUD where the CIs overlapped across all time periods. Tables S5 and S6 present the full parental AUD and DUD results in relation to timing of diagnosis.

### Sensitivity analyses

After partitioning the exposure into parental non-intoxication and intoxication SUD diagnoses registered prior birth, the overall association remained with statistically significant ORs for any ID and mild-unspecified ID using both exposures (Tables S7 and S8). The only difference was that the non-intoxication diagnoses predicted increased risk of moderate-severe ID (OR: 2.0 [1.7–2.3]), while no such effect was observed for intoxication diagnoses (OR: 1.0 [0.5–1.8]). In addition, when adjusting for parental life-time ID (Table S9) and exclusion of all cases with chromosomal disorders (Table S10) the overall results were in line with the main analysis with only slight reductions in ORs. As expected, the parental analysis found that if you had one parent with SUD prior to birth it was more likely that the second parent also had SUD prior to birth. Among the children with maternal SUD prior birth, 17.0% had a father with SUD registered prior birth compared to 1.2% in those who did not have a mother with SUD. Conversely, in those with a father with SUD prior birth, 8.9% also had a SUD mother compared to 0.6% in those without a SUD father (Χ^2^ = 25,340, p < 0.0001). Similar results were found for parental AUD and DUD (Full results reported in Tables S11, S12 and S13). Finally, we analysed putative cohort effects that may have affected our results. When including children whose parents were born prior to 1955 the cohort was larger (n = 2,416,388) but the results were similar as in the main analysis (Table S14). After subdividing the cohort into the oldest (born 1978–1990) and youngest (born 1991–2002) the rates of parental SUD and ID were slightly higher in the younger compared to the older cohort–but the relative risk estimates remained similar with overlapping CIs in both cohorts, suggestive of no significant cohort effects driving the overall association of parental SUD and ID (Tables S15 and S16). Finally, after excluding all individuals who died or emigrated during the study follow-up period, the results were identical as in the main analysis (Table S17).

## Discussion

The current study found that both maternal and paternal SUD diagnosed before offspring birth constitute a risk factor for the development of ID in offspring. The associations were clearly reduced but remained statistically significant after adjusting for multiple parental covariates, highlighting that the association is partly explained by socioeconomic factors and psychiatric comorbid disorders. Our results contribute to existing ID literature by replicating previous findings of maternal alcohol abuse as a risk factor for ID, while extending this finding to both maternal and paternal SUD. The observed association could be explained by shared genetic factors between SUD and ID, as well as environmental factors associated with parental SUD discussed below. The greatest risk increase for ID in offspring was observed when the parental AUD diagnosis was registered during pregnancy, while this association was not as evident for parental DUD, suggesting a specific toxic effect of alcohol intake during pregnancy, even though the absolute numbers of such cases were low rendering these conclusions uncertain.

Previous studies have found that children of parents with SUD are a vulnerable group, at increased risk for several adverse outcomes, including emotional, behavioural, and cognitive problems.^10^ The current study extends previous research by showing that parental SUD of both sexes constitute a risk factor also for the more severe cognitive deficit phenotype of ID. For maternal AUD this has been known for decades, given the established role of maternal alcohol intake during pregnancy in development of FASD,^16^^,^^20^^,^^21^ in which different degrees of ID are present.^14^ Our results are in line with two recent reviews on risk factors for ID, in which several prenatal maternal risk factors were found including maternal alcohol use during pregnancy,^17^^,^^18^ while our finding of paternal AUD and parental DUD is to the best of our knowledge a novel finding. Prevention efforts, education of health care staff and public health recommendations have for decades focused on mothers with AUD,^22^ while paternal AUD or other forms of parental SUD are rarely mentioned. The current study, however, highlights that paternal SUD represents at the population level an equally great risk factor for offspring ID. This suggests that SUD status in fathers also needs to be taken into consideration when developing prevention programs and diagnostic efforts targeting ID in children, irrespective of the underlying mechanism. Prevention programs offering SUD treatment to SUD parents who are planning to have children could possibly have a risk reducing effect on ID risk in offspring. Furthermore, awareness of the association between parental SUD and ID in children among health care professionals could also increase probability of early diagnosis and support for such at-risk children who develop ID.

The current study found that adjustment for sex, parental education and psychiatric co-morbidity consistently reduced the risk estimates, which corroborate previous findings of heightened risk of ID in males as well as in families with lower socioeconomic status and education and parental mental health problems.^23^ Given the observational nature of the current and previous studies, it remains difficult to discern through which mechanism such general risk factors relate to development of ID. While some observed risk factors could putatively exert a direct biological effect on neurodevelopment (e.g., substance abuse during pregnancy as discussed in the current study), other risk factors such as minority status and low socioeconomic status can also be understood as indices of a larger multifactorial complex of contributing multiple factors which can increase risk of neurodevelopmental adversity.^24^ Our results thus suggest that parents with lower socioeconomic status, SUD and comorbid mental disorders represent important risk factors when developing efforts for prevention and early detection of ID in children.

Heritability is defined as the proportion of phenotypic variance explained by genetic factors,^25^ and the observed association between parental SUD and offspring ID should be viewed in light of recent research that has shown that ID has a high heritability of 0.95, driven mainly by mild ID cases representing the majority of cases.^26^ Furthermore, Reichenberg and colleagues^27^ found that mild ID represents the end of the normally distributed phenotype of general cognitive ability, sharing familial factors with general cognitive ability in relatives. In contrast, severe ID was found to not share familial factors with general cognitive ability, suggesting an etiologically different causal mechanism, possibly due to de novo mutations.^8^^,^^27^^,^^28^ Previous studies have shown that parental SUD shares genetic factors with offspring general cognitive ability,^12^^,^^13^ and case control studies indicate that family history of SUD in healthy volunteers without substance-related problems is associated with specific cognitive deficits related to impulse control.^29^ One plausible hypothesis thus is that the observed association between parental SUD and ID, may be explained in part by shared genetic factors between SUD and milder forms of ID. This is also in line with the results from the timing analysis, where parental SUD increased the risk of mild ID at overall similar levels irrespective of when diagnosis was registered. To the best of our knowledge, it is not known whether SUD and ID do in fact share underlying genetic factors. Future quantitative genetic studies are needed however, to shed light on the relative importance of genetic versus environmental factors underlying the association between parental SUD and ID.

In the pregnancy analysis, we found that maternal SUD diagnosed before or during pregnancy increased risk for any form of ID in offspring, and was even higher compared to if diagnosis was registered after birth. Naturally in utero effects of substance intake are very difficult to study using observational data alone, but our results are in line with preclinical, clinical and epidemiological studies that have suggested that foetal exposure of alcohol, nicotine and illicit drugs including cocaine, stimulants, cannabis and opioids may exert negative effect on foetal neuronal development increasing risks of cognitive problems.^30^ It should be noted that smoking is highly prevalent among individuals with SUD.^31^ Studies of clinical SUD patients have reported high prevalence rates of current smoking around 80%,^32^ and meta analyses have suggested that cigarette consumption during pregnancy may be associated with negative effects on neurodevelopment in offspring.^33^^,^^34^ On the other hand, maternal smoking has also been shown to be an indicator of genetic or other environmental factors linked to offspring neurodevelopment.^35^^,^^36^ For instance, a recent study on Danish registry data using family based analyses suggested no causal role of maternal smoking on ID risk in offspring, but rather suggested the association was due to residual confounding factors.^37^ Our findings should thus be viewed in light of tobacco use constituting a possible confounding and/or exacerbating factor together with other substance use, with regards to the overall association between parental SUD and ID in offspring.

While those mothers receiving AUD diagnosis during pregnancy in our study were few (n = 276), and likely represent the most severe phenotypes of AUD, alcohol intake during pregnancy is not uncommon with a global prevalence of 9.8%.^38^ In Sweden, a study of approximately 1100 pregnant women who attended antenatal clinics reported their alcohol consumption anonymously found that approximately 30% drank alcohol.^39^ In addition, among those who drink alcohol during pregnancy, up to 40% also consume other addictive substances of which nicotine and cannabis were the most common,^40^ again highlighting the importance of considering polysubstance use in interpretation of our results. Specifically in our study, maternal AUD diagnosis registered during pregnancy conferred the greatest risk increase for all forms of ID in offspring while DUD diagnosed during pregnancy did not exhibit the same pattern. First, this suggests that alcohol may exert a substance-specific negative effect on foetal development in comparison to other drugs. This is in line with previous research which has established an association of maternal alcohol intake during pregnancy and FAS disorders,^14^ and extensive previous research showing negative impact of foetal ethanol exposure on brain development and cognitive functioning.^41^ We note that the OR of 5.5 for maternal AUD diagnosed during pregnancy was the highest of all estimates for offspring severe-moderate ID. Since severe cases of ID has been proposed to be due to de novo mutations,^27^^,^^28^ and ethanol has been shown to increase mutation rates through recruitment of error-prone polymerases,^42^ one possible interpretation is that a proportion of severe ID cases could be due to in utero alcohol toxic effects on foetal development. It should be noted that the number of parents and ID cases in the pregnancy analysis were very few, and the results should thus be interpreted with great caution.

Paternal AUD during pregnancy was associated with an elevated risk for ID in offspring, with higher OR compared to if AUD diagnosis was registered after birth, while this was not as evident for paternal DUD. We argue that the most probable interpretation of this is that paternal AUD likely represents a clinical marker of having a spouse with AUD not properly identified in the registries, with unidentified maternal AUD driving the association. This is supported by the significant spousal resemblance for AUD, i.e., if one parent has AUD it is more likely that the other parent also has the disorder which was supported in our sensitivity analysis as well as in prior research by Kendler and colleagues.^43^ A more speculative interpretation of the observed data however, is that paternal alcohol intake in adjacency to conception may cause de novo mutations directly affecting spermatogenesis, which in turn could influence ID risk. This is based on the assumption that AUD diagnosis registered during pregnancy reflect high alcohol intake also in the previous months before conception. Previous studies have found an association between alcohol consumption and reduced sperm quality in healthy men,^44^ and males with AUD exhibit hypogonadism and poor semen quality.^45^ To the best of our knowledge, it is not known whether heavy alcohol consumption directly causes de novo mutations in human spermatogenesis, even though preclinical studies have suggested that paternal ethanol consumption during preconception affects the male germline cells.^46^ It must, however, be noted that the research literature on this topic is scarce, and that this interpretation should be mainly viewed as hypothesis generating for future research.

The results of the current study have implications for prevention efforts, public health, medical education and social and health care services. The main finding of relevance for public health recommendations and education institutions is that at the population level, both maternal and paternal SUD represent a risk factor for development of ID in offspring. Irrespective of the underlying mechanism, this is important knowledge to consider when educating the public and medical professionals, including paediatric/psychiatric/neurological services assessing children with behavioural problems. In addition, it is important knowledge for addiction treatment services working with patients suffering from SUD to inform about the association of SUD and ID in offspring, as well as facilitating for children of SUD patients to receive neuropsychological evaluation if cognitive and behavioural problems are present. Also, children with ID need high levels of parental support and caregiving which is also likely compromised if parental substance use problems remain untreated, highlighting the need to facilitate evidence-based treatment for parents suffering from SUD. Finally, our results also highlight the need of maternity care services to identify SUD in pregnant women, as well as including the spouses for assessment and evaluation of substance related problems. This is of great importance especially during the pregnancy period, where diagnosed parental AUD conferred the highest risk of ID in offspring in the current study.

The results of the study should be viewed in light of some important limitations. First, registration of a SUD diagnosis likely represents substance-related problems of a more severe kind, and our findings are thus generalizable for more severe forms of SUD rather than milder forms of the disorder. Second, an inherent concern for all register-based studies is that our findings may be explained by some unmeasured covariate not included in the currently used national registries (e.g., smoking, infections, pre-term birth, environmental pollution). Thirdly, there is an inherent difficulty to assess onset of the disorder (in this case SUD) based on registry date in national registries. It is thus possible that, for instance, registration of a SUD diagnosis when the child is older still could mean that the SUD problems were evident earlier but did not result in hospitalisation or special care visit. Our results from the analysis of parental SUD prior birth and offspring ID should thus be viewed in light of this limitation i.e., the possibility of false negatives amongst the parents which may underestimate the observed risks. Fourth, Sweden has a long-standing alcohol culture and restrictive narcotic policy. The magnitude of the observed associations could thus differ in other countries where the consumption pattern of alcohol and illicit drugs differ markedly from Sweden. Fifth, information regarding which type of illicit drugs were used in the DUD group was not available for ICD-8 and ICD-9 but only in ICD-10 (from 1997 and onward). Since only a minority of parents in the current cohort could receive an ICD-10 diagnosis, our study can not answer the question of whether any type of DUD was associated with higher risk for ID. Future research with longer follow-up and complete information regarding substance diagnosis types can further elucidate this important research question. Finally, the current registry linkage did not include somatic diagnoses related to SUD for instance alcohol induced liver cirrhosis. Individuals with only such somatic SUD diagnoses were thus false negative and included in the unexposed group, which raises the possibility that our estimation of relative risk increase in fact could be an underestimation.

In summary, this study found that both mothers and fathers with SUD are at an increased risk of having children with ID. The underlying mechanism is not known but may involve shared genetic factors and environmental factors associated with parental SUD including alcohol intake during foetal development. These findings have implications for public health, healthcare and social services in suggesting that parental SUD of either parent is an important possibly modifiable risk factor to consider when developing prevention, diagnostics and treatment programs for children with ID.

## Contributors

Conceptualisation, study design and data interpretation were done by all authors. Data was analysed by LK RKH and PL with assistance by HL and AL. LK and RKH had access to and verified the study data. LK wrote the first draft of the manuscript and all authors participated in revising the manuscript.

## Data sharing statement

The study was performed by using data from the Swedish population registers. The Public Access to Information and Secrecy Act in Sweden prohibits us from making individual-level data publicly available.

## Declaration of interests

HL reports receiving grants from Shire/Takeda Pharmaceuticals; personal fees from and serving as a speaker for Medice, Shire/Takeda Pharmaceuticals and Evolan Pharma AB, all outside the submitted work. The other authors report no biomedical financial interests or potential conflicts of interests.
