# Supplementary material for: Parental substance use disorder and risk of intellectual disability in offspring in Sweden: a national register study
Source: eClinicalMedicine. 2023 Aug 30;63:102170. doi: 10.1016/j.eclinm.2023.102170 (PMC10480548; doi:10.1016/j.eclinm.2023.102170)
Supplement: Supplementary Tables [file mmc1.pdf]

Supplementary material

Title: Parental Substance Use Disorder and Risk of Intellectual Disability in Offspring

Authors: Lotfi Khemiri, Ralf Kuja-Halkola, Henrik Larsson, Agnieszka Butwicka, Magnus Tideman, Brian M. D'Onofrio, Antti Latvala and Paul Lichtenstein

National registries

The National Patient Register (NPR) includes registered diagnoses for hospitalizations (from 1973) and specialist outpatient visits (from 2001) until 2020 (*Ludvigsson JF, Andersson E, Ekbom A, et al. External review and validation of the Swedish national inpatient register. BMC Public Health 2011; 11: 450*). The Multi-Generation Register (*Ekbom A. The Swedish Multi-generation Register. Methods Mol Biol 2011; 675: 215–20*) includes information on biological parents allowing for identifying parental status and parental covariates. Information from the Migration Register, Total Population Register (*Ludvigsson JF, Almqvist C, Bonamy A-KE, et al. Registers of the Swedish total population and their use in medical research. Eur J Epidemiol 2016; 31: 125–36.*), and Cause of Death Register was used for creating the cohort and including covariates for the sensitivity analyses. The register-linkage has been approved by the Regional Ethical Review Board in Stockholm, Sweden.

Covariates

Offspring sex, birth year and mother's and father's age at birth of child was included as covariates in the main model. In the sensitivity analyses, we further adjusted for additional covariates: Highest level of parental education was extracted from the Longitudinal Integration Database for Health Insurance and Labour Market Studies (LISA) 1990-2019. Parental immigration status was extracted from the Migration Register and defined as a categorical variable as being born in Sweden or not. Psychiatric co-morbidity was extracted from the NPR and included psychotic disorders, bipolar disorder, depression and anxiety disorders as described in Table S3.

Table S1. Description of the substance use disorder (SUD) diagnoses, subdivided into alcohol use disorder (AUD) and drug use disorder (DUD), extracted from the National Patient Register to operationalize parental SUD.

	ICD 8	ICD 9	ICD 10
**SUD**	291<br>303<br>304	291<br>292<br>303<br>304<br>305<br>980	F10-F19 (except F17)<br>X41<br>X42<br>X45<br>X61<br>X62<br>X65<br>Y11<br>Y12<br>Y15<br>T40<br>T51
**AUD**	291<br>303	291<br>303<br>305A<br>980	F10<br>X45<br>X65<br>Y15<br>T51
**DUD**	304	292<br>304<br>305X	F11 – F19 (except F17)<br>X41<br>X42<br>X61<br>X62<br>Y11<br>Y12<br>T40

Table S2. Description of all outcome ICD diagnoses related to intellectual disability (ID) extracted from the National Patient Register, including which diagnoses were classified as mild-unspecific and moderate-severe, respectively.

	ICD 8	ICD 9	ICD 10
**Mild ID[a]**	310<br>311	317	F70
**Unspecified ID[a]**	315	319	F78<br>F79
**Severe-Moderate ID**	312<br>313<br>314	318	F71<br>F72<br>F73

a) Included in the category mild-unspecified ID

Table S3. Description of the ICD psychiatric diagnostic codes extracted from the National Patient Register to operationalize parental psychiatric co-morbidity.

	ICD 8	ICD 9	ICD 10
**Schizophrenia**	295	295	F20
**Bipolar disorder**	296	296 (except 296B)	F30-F31
**Depression**	300,4	296B<br>300E<br>311	F32 (except F32.3)<br>F33<br>F34<br>F38<br>F39
**Anxiety**	300 (except 300,4)	300 (except 300E)	F40-F42<br>F44<br>F45<br>F48

Table S4. Description of the risk estimates for all the covariates in the fully adjusted model (model 3) in the main analysis of parental substance use disorder (SUD) prior to childbirth as predictor of intellectual disability in offspring. Values are presented as odds ratios with 95% confidence intervals in brackets. Standard errors were adjusted for the clustering of siblings.

Full sample (n= 1,928,488)	**SUD in any parent**
**Birth year**	1·0 [1·0 - 1·0]
**Age at birth, mother**	1·0 [1·0 - 1·0]
**Age at birth, father**	1·0 [1·0 - 1·0]
**Sex (female)**	0·8 [0·7 - 0·8]
**Highest education, mother**	0·8 [0·7 - 0·8]
**Highest education, father**	0·9 [0·9 - 0·9]
**Immigrant, mother**	1·0 [0·9 - 1·0]
**Immigrant, father**	1·1 [1·1 - 1·2]
**Psychiatric co-morbidity, mother**	1·9 [1·7 - 2·1]
**Psychiatric co-morbidity, father**	1·6 [1·4 - 1·8]

Table S5. Different time periods of paternal and maternal alcohol use disorder (AUD) diagnosis registration as predictors of any intellectual disability (ID), severe-moderate ID and mild-unspecified ID using logistic regression modelling with adjustment for sex, birth year and age at birth in mother and father. Values are presented as odds ratios with 95% confidence intervals in brackets. Standard errors were adjusted for the clustering of siblings.

Full sample (n=1,940,820)	**Parental AUD before pregnancy (n=26,198)**	**Parental AUD during pregnancy (n=2,084)**	**Parental AUD after birth (n=157,253)**
**Any ID**			
No. cases (%)	807 (3·1)	78 (3·7)	3,069 (2·0)
OR	2·4 [2·2 – 2·5]	3·0 [2·4 – 3·8]	1·7 [1·6 – 1·8]
**Severe-moderate ID**			
No. cases (%)	159 (0·6)	20 (1·0)	671 (0·4)
OR	1·8 [1·6 – 2·2]	3·0 [1·9 – 4·7]	1·5 [1·3 – 1·6]
**Mild-unspecified ID**			
No. cases (%)	648 (2·5)	58 (2·8)	2,398 (1·5)
OR	2·5 [2·3 – 2·7]	3·0 [2·3 – 3·9]	1·8 [1·7 – 1·9]
	**Maternal AUD before pregnancy (n=8,933)**	**Maternal AUD during pregnancy (n=276)**	**Maternal AUD after birth (n=58,189)**
**Any ID**			
No. cases (%)	277 (3·1)	17 (6·2)	1,282 (2·2)
OR	2·3 [2·0 – 2·6]	5·0 [3·1 – 8·2]	1·9 [1·8 – 2·0]
**Severe-moderate ID**			
No. cases (%)	54 (0·6)	5 (1·8)	268 (0·5)
OR	1·8 [1·4 – 2·4]	5·5 [2·3 – 13·4]	1·5 [1·4 – 1·7]
**Mild-unspecified ID**			
No. cases (%)	223 (2·5)	12 (4·3)	1,014 (1·7)
OR	2·4 [2·1 – 2·8]	4·7 [2·6 – 8·3]	2·0 [1·8 – 2·1]
	**Paternal AUD before pregnancy (n=18,308)**	**Paternal AUD during pregnancy (n=1,836)**	**Paternal AUD after birth (n=107,360)**
**Any ID**			
No. cases (%)	574 (3·1)	63 (3·4)	2,030 (1·9)
OR	2·4 [2·2 – 2·7]	2·8 [2·2 – 3·6]	1·6 [1·6 – 1·7]
**Severe-moderate ID**			
No. cases (%)	112 (0·6)	16 (0·9)	461 (0·4)
OR	1·8 [1·5 – 2·2]	2·7 [1·7 – 4·5]	1·5 [1·3 – 1·6]
**Mild-unspecified ID**			
No. cases (%)	462 (2·5)	47 (2·6)	1,569 (1·5)
OR	2·6 [2·4 – 2·9]	2·8 [2·1 – 3·7]	1·7 [1·6 – 1·8]

Table S6. Different time periods of paternal and maternal drug use disorder (DUD) diagnosis registration as predictors of any intellectual disability (ID), severe-moderate ID and mild-unspecified ID using logistic regression modelling with adjustment for sex, birth year and age at birth in mother and father. Values are presented as odds ratios with 95% confidence intervals in brackets. Standard errors were adjusted for the clustering of siblings.

Full sample (1,940,820)	**Parental DUD before pregnancy (n=14,120)**	**Parental DUD during pregnancy (n=1,565)**	**Parental DUD after birth (n=79,569)**
**Any ID**			
No. cases (%)	399 (2·8)	46 (2·9)	1,929 (2·4)
OR	2·2 [1·9 – 2·4]	2·2 [1·6 – 2·9]	2·1 [2·0 – 2·2]
**Severe-moderate ID**			
No. cases (%)	81 (0·6)	9 (0·6)	389 (0·5)
OR	1·7 [1·4 – 2·2]	1·7 [0·9 – 3·4]	1·7 [1·5 – 1·8]
**Mild-unspecified ID**			
No. cases (%)	318 (2·3)	37 (2·4)	1,540 (1·9)
OR	2·3 [2·0 – 2·6]	2·3 [1·7 – 3·2]	2·2 [2·1 – 2·3]
	**Maternal DUD before pregnancy (n=5,803)**	**Maternal DUD during pregnancy (n=468)**	**Maternal DUD after birth (n=40,698)**
**Any ID**			
No. cases (%)	175 (3·0)	17 (3·6)	1,040 (2·6)
OR	2·3 [2·0 – 2·7]	2·7 [1·7 – 4·5]	2·1 [2·0 – 2·3]
**Severe-moderate ID**			
No. cases (%)	33 (0·6)	4 (0·9)	208 (0·5)
OR	1·7 [1·2 – 2·5]	2·5 [0·9 – 6·8]	1·7 [1·5 – 2·0]
**Mild-unspecified ID**			
No. cases (%)	142 (2·4)	13 (2·8)	832 (2·0)
OR	2·5 [2·1 – 3·0]	2·8 [1·6 – 4·8]	2·2 [2·1 – 2·4]
	**Paternal DUD before pregnancy (n=9,380)**	**Paternal DUD during pregnancy (n=1,185)**	**Paternal DUD after birth (n=42,860)**
**Any ID**			
No. cases (%)	247 (2·6)	33 (2·8)	1,034 (2·4)
OR	2·0 [1·7 – 2·3]	2·1 [1·5 – 2·9]	2·0 [1·9 – 2·1]
**Severe-moderate ID**			
No. cases (%)	51 (0·5)	7 (0·6)	205 (0·5)
OR	1·6 [1·2 – 2·2]	1·8 [0·9 – 3·8]	1·6 [1·4 – 1·9]
**Mild-unspecified ID**			
No. cases (%)	196 (2·1)	26 (2·2)	829 (1·9)
OR	2·1 [1·8 – 2·4]	2·1 [1·4 – 3·1]	2·1 [2·0 – 2·3]

Table S7. Parental substance use disorder (SUD) before birth, but excluding all intoxication diagnoses, as predictors of any intellectual disability (ID). Values are presented as odds ratios with 95% confidence intervals in brackets. Standard errors were adjusted for the clustering of siblings.

Full sample (n=1,940,820)	**SUD (no intoxication) in any parent**	**Maternal SUD (no intoxication)**	**Paternal SUD (no intoxication)**
**Any ID**	2·4 [2·2 - 2·6]	2·6 [2·3 - 2·9]	2·3 [2·1 - 2·5]
**Severe-moderate ID**	2·0 [1·7 - 2·3]	2·0 [1·6 - 2·6]	1·9 [1·6 - 2·2]
**Mild-unspecified ID**	2·5 [2·3 - 2·7]	2·7 [2·4 - 3·1]	2·4 [2·2 - 2·7]

Table S8. Parental substance use disorder (SUD) before birth, but including only the intoxication diagnoses, as predictors of any intellectual disability (ID). Values are presented as odds ratios with 95% confidence intervals in brackets. Standard errors were adjusted for the clustering of siblings.

Full sample (n=1,940,820)	**SUD (only intoxication) in any parent**	**Maternal SUD (only intoxication)**	**Paternal SUD (only intoxication)**
**Any ID**	2·0 [1·6 - 2·5]	1·7 [1·2 - 2·3]	2·3 [1·7 - 3·2]
**Severe-moderate ID**	1·0 [0·5 - 1·8]	0·9 [0·3 - 2·2]	1·0 [0·4 - 2·6]
**Mild-unspecified ID**	2·2 [1·8 - 2·8]	1·9 [1·3 - 2·6]	2·7 [1·9 - 3·8]

Table S9. Parental substance use disorder (SUD), including maternal and paternal SUD before childbirth, as predictors of any intellectual disability (ID) with adjustment for any parental ID diagnosis. Values are presented as odds ratios with 95% confidence intervals in brackets. Standard errors were adjusted for the clustering of siblings.

Full sample (n=1,940,820)	**SUD in any parent**	**Maternal SUD**	**Paternal SUD**
**Any ID**	2·0 [1·9 – 2·2]	2·0 [1·8 – 2·2]	2·0 [1·9 – 2·2]
**Severe-moderate ID**	1·7 [1·4 – 1·9]	1·6 [1·3 – 2·0]	1·6 [1·4 – 1·9]
**Mild-unspecified ID**	2·2 [2·0 – 2·3]	2·1 [1·8 – 2·3]	2·2 [2·0 – 2·4]

Table S10. Parental substance use disorder (SUD), including maternal and paternal SUD before childbirth, as predictors of any intellectual disability (ID) but exclusion of individuals (n=5,289) with any diagnosis of chromosomal disorders (ICD 9: 758; ICD-10: Q90-Q99). Values are presented as odds ratios with 95% confidence intervals in brackets. Standard errors were adjusted for the clustering of siblings.

Full sample (n= 1,935,531)	**SUD in any parent**	**Maternal SUD**	**Paternal SUD**
**Any ID**	2·4 [2·2 – 2·6]	2·4 [2·1 – 2·6]	2·4 [2·2 – 2·6]
**Severe-moderate ID**	2·0 [1·7 – 2·3]	2·0 [1·6 – 2·5]	1·9 [1·6 – 2·3]
**Mild-unspecified ID**	2·5 [2·3 – 2·7]	2·4 [2·2 – 2·7]	2·5 [2·3 – 2·7]

Table S11. Sensitivity analysis of association of maternal and paternal substance use disorder (SUD) prior to child birth. Frequencies of the co-parents SUD status is reported, and values in brackets are 95% confidence intervals. Chisquare tests of independence were performed to compare the two categorical variables.

Full sample (n=1,940,820)	**Mother without SUD n = 1,927,238**	**Mother with SUD n = 13,582**	**Statistical significance**
**Father with SUD**<br>**No.**<br>**% within each category of mothers**	 23,828<br>1·2 [1·2 - 1·3]	 2,314<br>17·0 [16·4 - 17·7]	 X^2 = 25,340, p <0·0001
	**Father without SUD n = 1,914,678**	**Father with SUD n = 26,142**	
**Mother with SUD**<br>**No.**<br>**%, within each category of fathers**	 11,268<br>0·6 [0·6 - 0·6]	 2,314<br>8·9 [8·5 - 9·2]	

Table S12. Sensitivity analysis of association of maternal and paternal alcohol use disorder (AUD) prior to child birth. Frequencies of the co-parents AUD status is reported, and values in brackets are 95% confidence intervals. Chisquare tests of independence were performed to compare the two categorical variables.

Full sample (n=1,940,820)	**Mother without AUD n = 1,931,618**	**Mother with AUD n = 9,202**	**Statistical significance**
**Father with AUD**<br>**No.**<br>**% within each category of mothers**	 19,043<br>1·0 [1·0 - 1·0]	 1,070<br>11·6 [11·0 - 12·3]	 X^2 = 10,113, p <0·0001
	**Father without AUD n = 1,920,707**	**Father with AUD n = 20,113**	
**Mother with AUD**<br>**No.**<br>**%, within each category of fathers**	 8,132<br>0·4 [0·4 - 0·4]	 1,070<br>5·3 [5·0 - 5·6]	

Table S13. Sensitivity analysis of association of maternal and paternal drug use disorder (DUD) prior to child birth. Frequencies of the co-parents DUD status is reported, and values in brackets are 95% confidence intervals. Chisquare tests of independence were performed to compare the two categorical variables.

Full sample (n=1,940,820)	**Mother without DUD n = 1,934,561**	**Mother with DUD n = 6,259**	**Statistical significance**
**Father with DUD**<br>**No.**<br>**% within each category of mothers**	 9,391<br>0·5 [0·5 - 0·5]	 1,149<br>18·4 [17·4 - 19·3]	 X^2 = 36,895, p <0·0001
	**Father without DUD n = 1,930,280**	**Father with DUD n = 10,540**	
**Mother with DUD**<br>**No.**<br>**%, within each category of fathers**	 5,110<br>0·3 [0·3 - 0·3]	 1,149<br>10·9 [10·3 - 11·5]	

Table S14. Parental substance use disorder (SUD), including maternal and paternal SUD before childbirth, as predictors of any intellectual disability (ID) without any restrictions regarding parental birth year. Values are presented as odds ratios with 95% confidence intervals in brackets. Standard errors were adjusted for the clustering of siblings.

Full sample (n= 2,416,388)	**SUD in any parent**	**Maternal SUD**	**Paternal SUD**
**Any ID**	2·4 [2·3 – 2·6]	2·5 [2·3 – 2·7]	2·4 [2·2 – 2·6]
**Severe-moderate ID**	1·9 [1·7 – 2·2]	2·0 [1·6 – 2·4]	1·9 [1·6 – 2·2]
**Mild-unspecified ID**	2·6 [2·4 – 2·8]	2·6 [2·4 – 2·9]	2·6 [2·4 – 2·8]
	**AUD in any parent**	**Maternal AUD**	**Paternal AUD**
**Any ID**	2·6 [2·4 – 2·8]	2·6 [2·3 – 2·9]	2·6 [2·4 – 2·8]
**Severe-moderate ID**	2·0 [1·7 – 2·3]	1·9 [1·5 – 2·5]	2·0 [1·7 – 2·3]
**Mild-unspecified ID**	2·8 [2·6 – 3·0]	2·8 [2·5 – 3·2]	2·8 [2·6 – 3·1]
	**DUD in any parent**	**Maternal DUD**	**Paternal DUD**
**Any ID**	2·2 [2·0 – 2·4]	2·4 [2·1 – 2·8]	2·0 [1·8 – 2·3]
**Severe-moderate ID**	1·8 [1·5 – 2·2]	1·9 [1·4 – 2·6]	1·7 [1·4 – 2·2]
**Mild-unspecified ID**	2·3 [2·1 – 2·6]	2·6 [2·2 – 3·0]	2·1 [1·8 – 2·4]

Table S15. Prevalence rates of parental SUD prior to child birth and intellectual disability (ID) of different severity in the oldest (born 1978-1990) and youngest (born 1991 - 2002) cohort participants. Values are presented as frequencies with 95% confidence intervals.

	**Older cohort (Born 1978-1990) n = 798,122**	**Younger cohort (Born 1991-2002) n = 1,142,698**
**Parental SUD prior to child birth**		
No.	12,991	24,419
%	1·6 [1·6 - 1·7]	2·1 [2·1 - 2·2]
**Any ID**		
No.	7,875	16,403
%	1·0 [1·0 - 1·0]	1·4 [1·4 - 1·5]
**Severe-moderate ID**		
No.	2,095	4,020
%	0·3 [0·3 - 0·3]	0·4 [0·3 - 0·4]
**Mild-unspecified ID**		
No.	5,780	12,383
%	0·7 [0·7 - 0·7]	1·1 [1·1 - 1·1]

Table S16. Parental substance use disorder (SUD) before childbirth as predictors of intellectual disability (ID) in the oldest (born 1978-1990) and youngest (born 1991 - 2002) cohort participants. Values are presented as odds ratios with 95% confidence intervals in brackets. Standard errors were adjusted for the clustering of siblings.

	**Parental SUD in older cohort (Born 1978-1990) n = 12,991**	**Parental SUD in younger cohort (Born 1991-2002) n = 24,419**
**Any ID**	2·6 [2·3 - 2·9]	2·2 [2·1 - 2·4]
**Severe-moderate ID**	2·1 [1·7 - 2·7]	1·8 [1·5 - 2·2]
**Mild-unspecified ID**	2·8 [2·4 - 3·2]	2·3 [2·2 - 2·6]

Table S17. Parental substance use disorder (SUD) before childbirth as predictors of intellectual disability (ID) after excluding all individuals who died (n=12,491) or emigrated (84,996) during the follow-up period. Values are presented as odds ratios with 95% confidence intervals in brackets. Standard errors were adjusted for the clustering of siblings.

Full sample (n= 1,843,564)	**SUD in any parent**	**Maternal SUD**	**Paternal SUD**
**Any ID**	2·3 [2·2 - 2·5]	2·3 [2·1 - 2·5]	2·3 [2·1 - 2·5]
**Severe-moderate ID**	1·9 [1·7 - 2·2]	1·9 [1·5 - 2·4]	1·8 [1·6 - 2·2]
**Mild-unspecified ID**	2·4 [2·2 - 2·6]	2·4 [2·1 - 2·7]	2·4 [2·2 - 2·6]
